# Efficacy and Safety of Daprodustat for Anemia Therapy in Chronic Kidney Disease Patients: A Systematic Review and Meta-Analysis

**DOI:** 10.3389/fphar.2020.573645

**Published:** 2021-01-12

**Authors:** Qiyan Zheng, Yahui Wang, Huisheng Yang, Luying Sun, Xinwen Fu, Ruojun Wei, Yu Ning Liu, Wei Jing Liu

**Affiliations:** ^1^ Renal Research Institution of Beijing University of Chinese Medicine, Beijing, China; ^2^ Key Laboratory of Chinese Internal Medicine of Ministry of Education and Beijing, Dongzhimen Hospital Affiliated to Beijing University of Chinese Medicine, Beijing, China; ^3^ Institute of Acupuncture and Moxibustion, China Academy of Chinese Medical Sciences, Beijing, China; ^4^ Institute of Nephrology, and Zhanjiang Key Laboratory of Prevention and Management of Chronic Kidney Disease, Guangdong Medical University, Zhanjiang, China

**Keywords:** daprodustat, anemia, chronic kidney disease, systematic review, meta-analysis

## Abstract

**Objective:** Daprodustat is a novel oral agent in treating anemia of chronic kidney disease (CKD), and several clinical trials have been conducted to compare daprodustat with recombinant human erythropoietin (rhEPO) or placebo. Our systematic review aimed to investigate the efficacy and safety of daprodustat for anemia treatment in both dialysis-dependent (DD) and non-dialysis-dependent (NDD) patients.

**Methods:** Six databases were searched for randomized controlled trials (RCTs) reporting daprodustat vs. rhEPO or placebo for anemia patients in CKD. The outcome indicators were focused on hemoglobin (Hb), ferritin, transferrin saturation (TSAT), total iron-binding capacity (TIBC), vascular endothelial growth factor (VEGF), and serious adverse events (SAEs).

**Results:** Eight eligible studies with 1,516 participants were included. For both NDD and DD patients, changes in Hb levels from baseline were significantly higher in daprodustat group than that in the placebo (mean difference (MD) = 1.73, [95% confidence interval (CI), 0.34 to 3.12], *p* = 0.01; MD = 1.88, [95% CI, 0.68 to 3.09], *p* = 0.002; respectively), and there was no significant difference between daprodustat and rhEPO group (MD = 0.05, [95% CI, −0.49 to 0.59], *p* = 0.86; MD = 0.12, [95% CI, −0.28 to 0.52], *p* = 0.55; respectively). The indexes of iron metabolism were improved significantly in the daprodustat group compared to placebo- or rhEPO-treated patients, while there was no similar change in terms of TSAT for DD patients. Furthermore, no trend of increasing plasma VEGF was observed in daprodustat-treated subjects. As for safety, there was no significant difference in the incidence of SAEs between daprodustat and placebo treatment, while the incidence of SAEs in the daprodustat group was significantly lower than that in the rhEPO group.

**Conclusion:** Daprodustat was efficacious and well tolerated for anemia in both NDD and DD patients in the short term based on current RCTs. And daprodustat may become an effective alternative for treatment of anemia with CKD. Since the application of daprodustat is still under exploration, future researches should consider the limitations of our study to evaluate the value of daprodustat.

## Introduction

Chronic kidney disease (CKD), as a major global health problem, is estimated to affect 8–16% of adult populations globally (Stanifer et al., 2016; Chen et al., 2019). Anemia, which is frequently observed in CKD, is not only a highly prevalent risk factor for many adverse events in CKD patients but also contributes to the progression of CKD and increases morbidity and mortality (Hörl, 2013; Cases et al., 2018). The causes of anemia in CKD patients are multi-factorial, including deficiency in erythropoietin (EPO), reduced iron availability, and inflammation (Babitt and Lin, 2012; Zumbrennen-Bullough and Babitt, 2014). Current treatments for anemia in CKD mainly require recombinant human erythropoietin (rhEPO) or its analogs (erythropoiesis stimulating agents [ESAs]) and iron supplementation (intravenous and/or oral) (KDIGO Clinical Practice Guideline Working Group, 2012; US Food and Drug Administration, 2011; Shepshelovich et al., 2016). While these therapies can greatly benefit patients by improving their quality of life and reducing the need for blood transfusions (Finkelstein et al., 2009; Parfrey et al., 2009), each has significant limitations, such as the inconvenience of subcutaneous injection that impact its use. Therefore, safer and more effective drugs are continuously being developed by a number of pharmaceutical companies for treatment of anemia in CKD patients.

Daprodustat (GSK1278863) is an orally active, small-molecule hypoxia-inducible factor (HIF) prolyl hydroxylase inhibitor (PHI) and belongs to an emerging new class of agents being developed for anemia in CKD in both non-dialysis dependent (NDD) and dialysis-dependent (DD) patients (Brigandi et al., 2016; Holdstock et al., 2016). Daprodustat can inhibit HIF-prolyl hydroxylase domain enzymes (PHD1, PHD2, and PHD3), resulting in the accumulation of HIF-α transcription factor and increased expression of HIF-responsive genes and inducing responses to hypoxia under normoxic conditions (Gupta and Wish, 2017). Increased expression of HIF-responsive genes promotes the synthesis of related proteins, such as EPO and proteins involved in iron uptake, mobilization, and transport, which lead to increased erythropoiesis and elevation of circulating Hb levels (Haase, 2013a). Compared with agents commonly used in treating anemia in CKD patients, daprodustat has certain potential advantages, such as avoiding the risk of supraphysiologic EPO levels and improving iron availability, which may reduce the need for iron replacement therapy (Lenihan and Winkelmayer, 2016). Although a pervious meta-analysis (Xie et al., 2018) showed that daprodustat could improve Hb without increasing adverse events in CKD patients, this study only included randomized controlled trials (RCTs) that compared with daprodustat and placebo but not daprodustat vs. rhEPO, and the evidence for efficacy and safety of daprodustat in two distinct groups of NDD patients and DD patients is lacking. In addition, several new clinical trials have been conducted to compare daprodustat with rhEPO or placebo for the treatment of anemia in dialyzed or non-dialyzed patients with CKD. Therefore, our systematic review aimed to investigate the efficacy and safety of daprodustat therapy for anemia in CKD in both NDD and DD patients, based on current phase 2 and phase 3 randomized controlled trial (RCT) results, and to provide evidence-based medicine for the clinical application of daprodustat.

## Methods

The review complies with the Preferred Reporting Items for Systematic Reviews and Meta-Analyses (PRISMA) guidelines (Moher et al., 2009) (Supplementary Table S1)

### Data Sources and Search Strategy

To identify potentially eligible studies, we searched PubMed, embase, Cochrane Library, Web of Science, ClinicalTrials.gov, and SinoMed databases from inception until March 2020 for clinical trials investigating daprodustat for anemia in patients with CKD in adults. Additional studies were searched in the reference lists of all identified publications, including relevant meta-analyses and systematic reviews.

### Inclusion Criteria

We included clinical studies that satisfied the following criteria: 1) type of study limited to randomized controlled trials (RCTs); 2) participants in the included studies were anemia with CKD (KDIGO Clinical Practice Guideline Working Group, 2012); 3) daprodustat as an intervention arm (the dosage of daprodustat were not restricted); 4) the control arm receiving other ways to contrast with daprodustat (included rhEPO or placebo); 5) the outcome indicators were focused on: 1) Hb, ferritin, transferrin saturation (TSAT), total iron-binding capacity (TIBC) and vascular endothelial growth factor (VEGF) (changes of five outcomes levels from baseline), 2) serious adverse events (SAEs).

### Data Extraction

Two investigators (XW. Fu and RJ. Wei) independently extracted data from included studies using a standardized form. Any disagreement was resolved by consensus between the reviewers or adjudication with a third party (HS. Yang), if necessary. Data extracted included study characteristics (first author, publication year, country, single or multi-center, sample size, intervention and control, the period of treatment, and duration of follow-up), characteristics of patients (inclusion criteria, background treatments, mean age, the proportion of men, the baseline of BMI, baseline of hemoglobin levels, and baseline of iron metabolism indices), reported outcomes (hemoglobin, ferritin, transferrin saturation, TIBC, VEGF, and SAEs), and information on methodology.

### Quality Assessment

The risk of bias of RCTs was assessed using the Cochrane Collaboration’s tool (Sterne et al., 2019). The included trials were graded as low quality, high quality, or moderate quality based on the following criteria: 1) trials were considered low quality if either randomization or allocation concealment was assessed as a high risk of bias, regardless of the risk of other items; 2) trials were considered high quality when both randomization and allocation concealment were assessed as low risk of bias, and all other items were assessed as low or unclear risk of bias; 3) trials were considered moderate quality if they did not meet criteria for high or low risk. Two investigators (HS. Yang and YH. Wang) independently coded the risk of bias for each included study, and discrepancies were discussed with a third party and resolved by consensus.

### Statistical Analyses

All analyses were performed using the Review Manager (RevMan) software version 5.3 Risk ratio (RR) and mean difference (MD) with 95% confidence interval (95% CI) of the outcomes were calculated as the effect measure. Heterogeneity was examined using a chi-squared test with Cochrane’s Q and I^2^ statistics. A fixed-effect (FE) model was used if I^2^ < 30%; otherwise, the random-effect (RE) model was used. Publication bias was tested by the visual inspection of the funnel plots. When a few studies are included in the analysis, the power of the tests is too low; therefore, publication bias was only examined if more than 10 study comparisons were included in the analysis (Shuster, 2011).

## Results

### Study Characteristics

**FIGURE 1:**
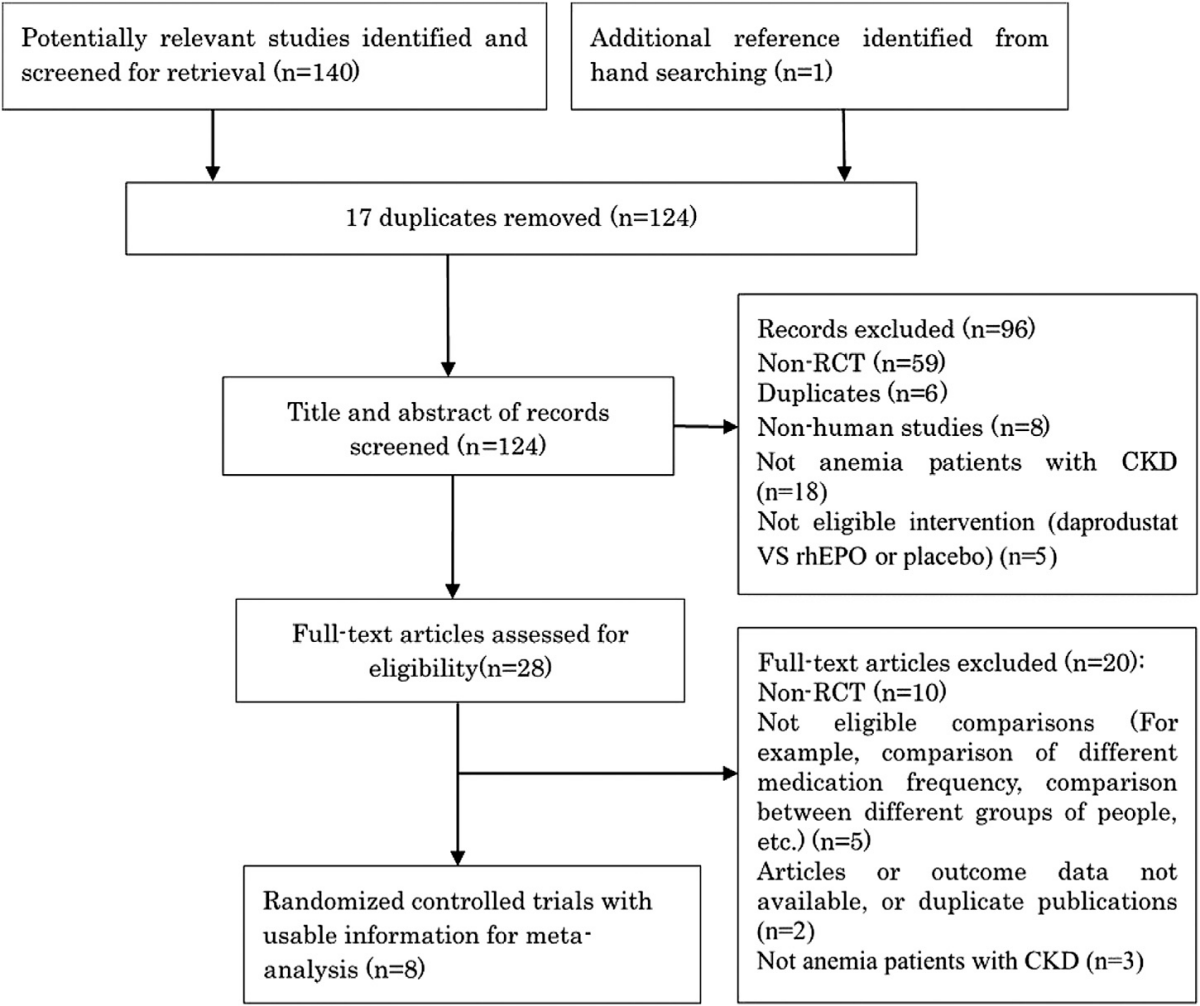
Flow chart of literature search and selection.

Our initial electronic database search captured 141 potential articles. Next, 124 titles and abstracts were screened, leaving 28 articles for full-text screening. Finally, eight eligible manuscripts (Brigandi et al., 2016; Holdstock et al., 2016; Akizawa et al., 2017; Bailey et al., 2019; Holdstock et al., 2019; Meadowcroft et al., 2019; GlaxoSmithKline, 2016a; GlaxoSmithKline, 2016b) (1,514 participants) evaluated the efficacy and safety of daprodustat for anemia in CKD patients Figure 1 illustrates the screening process. Characteristics of the included studies are summarized in Table 1.

**TABLE 1:** Characteristics of the included studies in the Meta-analysis.

Study ID	Location	Single/multicenter	Patients type	Age (year) mean ± SD	Baseline Hb (g/dl)	Interventions	No	Dosage of medication	Study duration	Clinical trial
Holdstock2016	United States	Multicenter	NDD patients	69.2 ± 11.0	9.91 ± 0.57	Placebo	18	Orally once-daily	4 weeks	Phase 2a
				71.3 ± 11.3	10.08 ± 0.72	Daprodustat	54	0.5, 2, 5 mg once-daily		
			DD patients	64.2 ± 12.8	10.89 ± 0.52	rhEPO	20	Not mentioned	4 weeks	Phase 2a
				55.6 ± 17.9	10.80 ± 0.61	Daprodustat	62	0.5, 2, 5 mg once-daily		
Brigandi et al., 2016	Australia, New Zealand, India, Russia	Multicenter	NDD patients	54.8 ± 17.26	-	Placebo	9	Orally once-daily	28 days	Phase 2a
				62.1 ± 12.38	-	Daprodustat	61	10, 25, 50, 100 mg once-daily		
			DD patients	57.2 ± 7.03	-	Placebo	6	Orally once-daily	28 days	Phase 2a
				50.0 ± 12.01	-	Daprodustat	31	10, 25 mg once-daily		
Meadowcroft et al., 2019	Global study	Multicenter	DD patients	59.7 ± 18.7	10.6 ± 0.94	rhEPO	39	Placebo qd for 4w + rhEPO (epoetin or their biosimilars, or darbepoetin alfa) for the remaining 20 weeks	24 weeks	Phase 2
				59.6 ± 13.3	10.4 ± 0.66	Daprodustat	177	a fixed-dose of 4, 6, 8 10,12 mg for 4 weeks+the need for dose adjustments was evaluated every 4 weeks		
Holdstock et al., 2019	Global study	Multicenter	NDD patients	65.4 ± 13.6	10.1 ± 0.67	rhEPO	80	rhEPO (epoetins or their biosimilars or darbepoetin), chose the rhEPO dose to achieve and maintain Hb levels within the target range	24 weeks	Phase 2
				66.5 ± 12.78	10.0 ± 0.77	Daprodustat	170	rhEPO-naı¨ve: once-daily daprodustat (1, 2 or 4 mg daily depending on baseline Hb); rhEPO-user:daprodustat 2 mg daily (fixed 4w,later make dose adjustment to achieve Hb target range)		

Study ID	Location	Single/multicenter	Patients type	Age (year) mean ± SD	Baseline Hb (g/dl)	Interventions	No	Dosage of medication	Study duration	Clinical trial
Akizawa et al., 2017	Japan	Multicenter	DD patients	63.4 ± 8.98	8.5–10.5	Placebo	19	Orally once daily	4 weeks	Phase 2
				62.4 ± 9.7	-	Daprodustat	97	4, 6, 8, 10 mg orally once daily		
Christine 2019	Russia,Spain,United States,Canada,Germany	Multicenter	DD patients	60.9 ± 13.3	10.6 ± 0.75	Placebo	19	Not mentioned, placebo	29 days	Phase 2
				64.1 ± 15.0	10.6 ± 0.69	Daprodustat	84	10, 15, 25, 30 mg three times weekly		
Tadao 2019	Japan	Multicenter	DD patients	63.5 ± 10.54	10.82 ± 0.73	Darbepoetin alfa	135	IV darbepoetin alfa injection (10,15,20,30,40, and 60 μg [ug] as recommended) once weekly	52 weeks	Phase 3
				64.1 ± 10.3	10.94 ± 0.77	Daprodustat	136	Oral daprodustat tablets (1, 2, 4, 6, 8, 12, 18, and 24 mg [mg] as recommended) once daily		
NCT02791763	Japan	Multicenter	NDD patients	70.3 ± 9.09	10.68 ± 1.07	Epoetin beta	150	Subcutaneous epoetin beta pegol (25, 50, 75, 100, 150, 200, or 250 μg [µg] as recommended) dose once every 2 or 4 weeks	52 weeks	Phase 3
				68.2 ± 11.57	10.48 ± 1.14	Daprodustat	149	Oral daprodustat (1, 2, 4, 6, 8, 12, 18, or 24 mg [mg] as recommended) dose once daily		

**Note:** DD, dialysis-dependent; NDD, non-dialysis dependent; rhEPO, recombinant human erythropoietin; IV, intravenous.

### Evaluation of the Risk of Bias of Selected Studies

**FIGURE 2:**
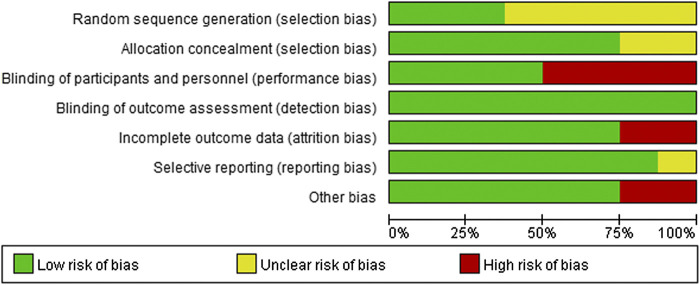
Risk-of-bias summary of included randomized trials using the Cochrane risk-of-bias tool.

The risk of bias involving the included randomized trials was assessed using the Cochrane Risk-of-Bias tool. The outcome was that all studies were judged to be at low risk of bias, because the indicators included in this study were relatively objective. Most RCTs were assessed as having low risk of bias for allocation concealment, blinding of participants and personnel, incomplete outcome data, selective reporting, and other sources of bias. Most RCTs were assessed as having unclear risk of bias for sequence generation, considering that detailed information was not provided. However, four studies (Holdstock et al., 2019; Meadowcroft et al., 2019; GlaxoSmithKline, 2016a; GlaxoSmithKline, 2016b) had high risk of bias for blinding of participants and personnel, because these could not be done. The risk of assessment bias of the included trials is shown in Figure 2.

### Meta-Analysis

#### Changes in Hemoglobin Levels From Baseline (△Hemoglobin)

**FIGURE 3:**
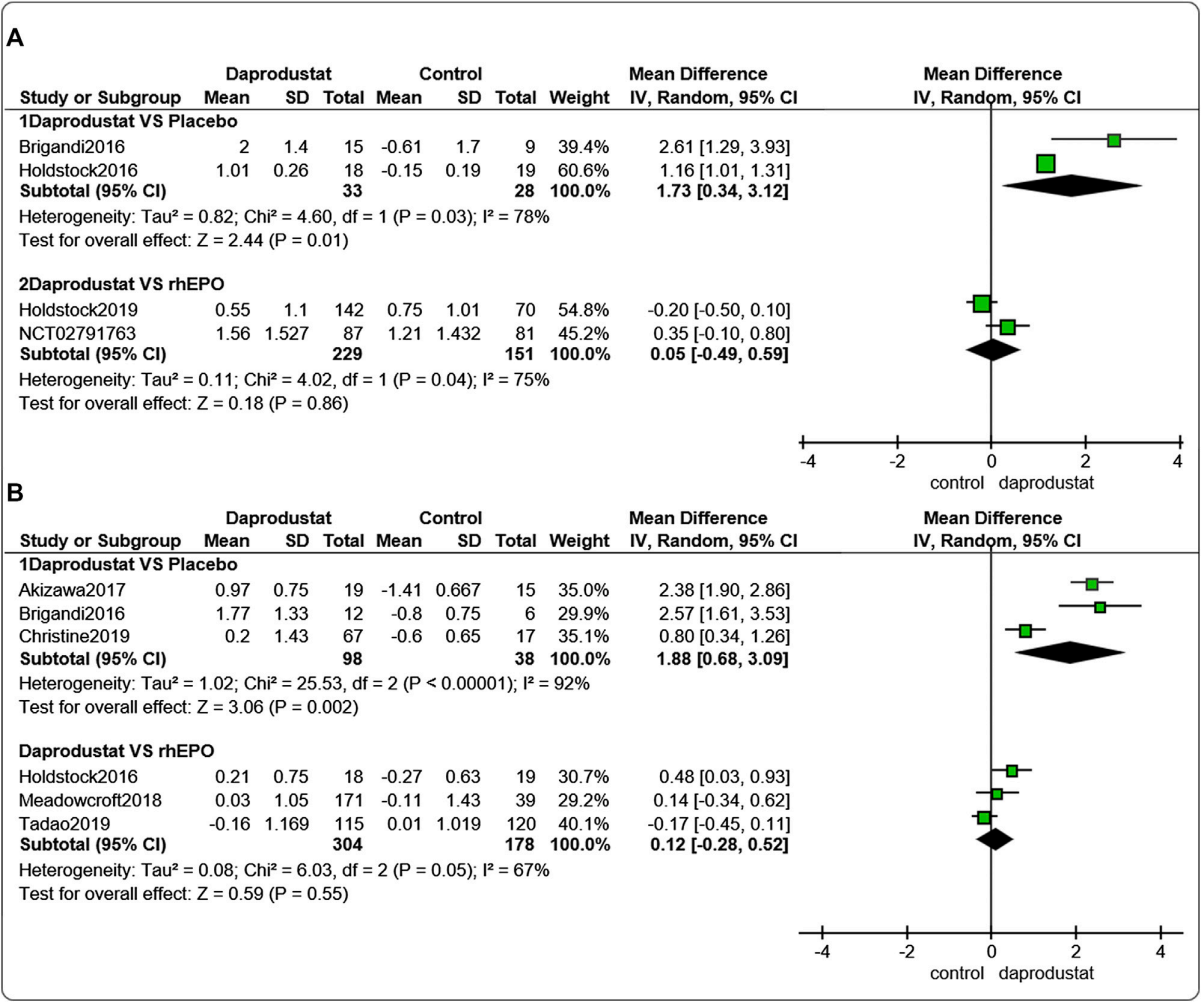
The meta-analysis results of daprodustat for △Hb. **(A)** The pooled results for NDD patients; **(B)** The pooled results for DD patients.

Four studies (Brigandi et al., 2016; Holdstock et al., 2016; Holdstock et al., 2019; GlaxoSmithKline, 2016a) involving 441 participants reported △Hb levels for NDD patients. Among them, two trails were comparisons between daprodustat and placebo, and two studies were comparisons between daprodustat and rhEPO. Six studies (Brigandi et al., 2016; Holdstock et al., 2016; Akizawa et al., 2017; Bailey et al., 2019; Meadowcroft et al., 2019; GlaxoSmithKline, 2016b) involving 618 participants reported △Hb levels for DD patients. Among these, three trials involved comparisons between daprodustat and placebo, and three studies were comparisons between daprodustat and rhEPO. The pooled results showed that for both NDD patients (Figure 3A) and DD patients (Figure 3B), △Hb levels were significantly higher in the daprodustat group than that in placebo cohort (MD = 1.73, [95% CI, 0.34 to 3.12], *p* = 0.01; MD = 1.88, [95% CI, 0.68 to 3.09], *p* = 0.002; respectively), but there was no significant difference between daprodustat and rhEPO in terms of changes in Hb levels (MD = 0.05, [95% CI, −0.49 to 0.59], *p* = 0.86; MD = 0.12, [95% CI, −0.28 to 0.52], *p* = 0.55; respectively).

#### Changes in Ferritin Levels From Baseline (△ferritin)

**FIGURE 4:**
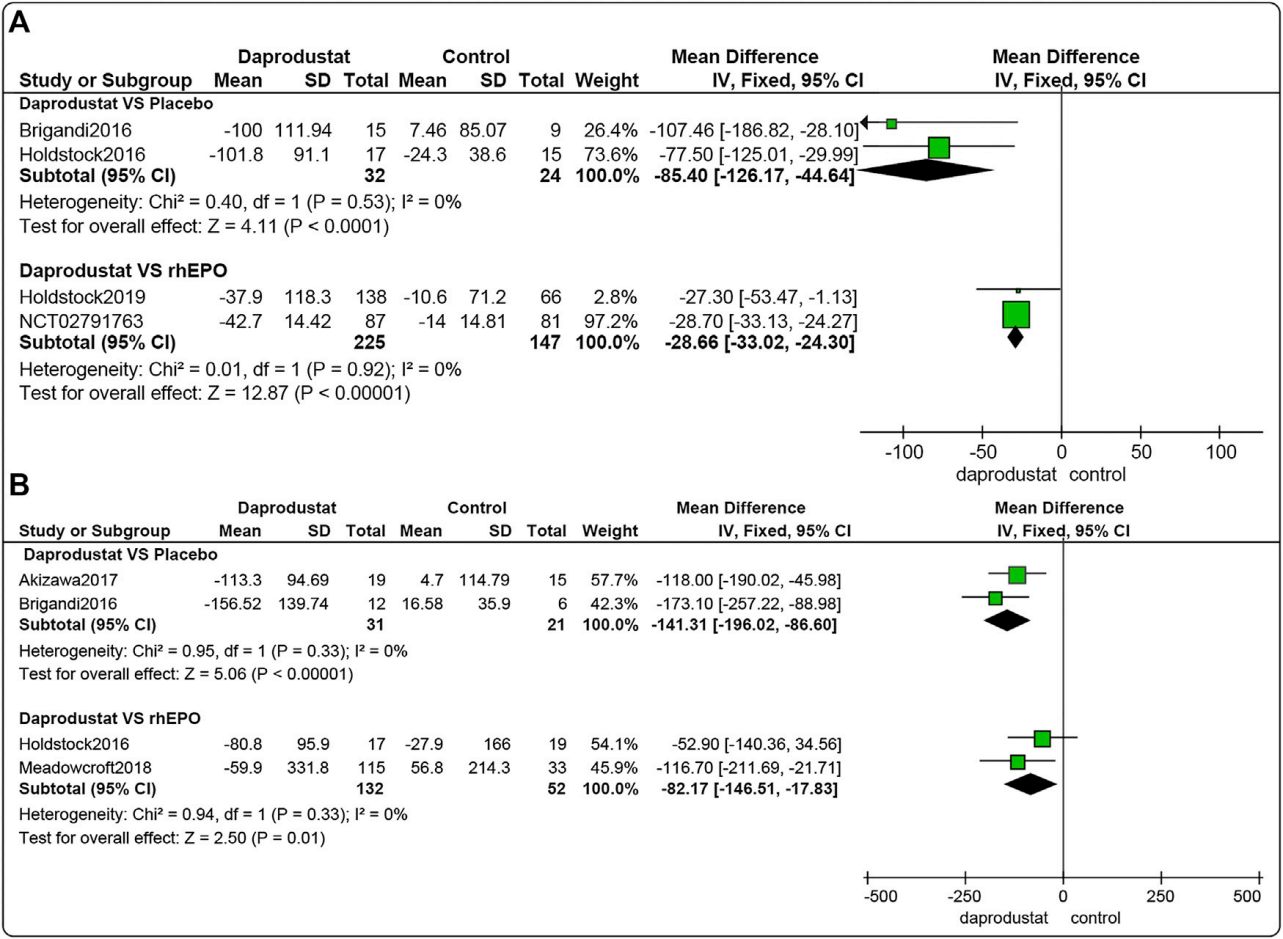
The meta-analysis results of daprodustat for △ferritin. **(A)** The pooled results for NDD patients; **B**: The pooled results for DD patients.

Four studies (Brigandi et al., 2016; Holdstock et al., 2016; Holdstock et al., 2019; GlaxoSmithKline, 2016a) involving 428 participants reported △ferritin levels for NDD patients, and four studies (Brigandi et al., 2016; Holdstock et al., 2016; Akizawa et al., 2017; Meadowcroft et al., 2019), including 236 participants, reported △ferritin levels in DD patients. Among these, for both NDD patients and DD patients, two trials involved comparisons between daprodustat and placebo, and two studies compared daprodustat and rhEPO. Pooled results showed that for NDD patients (Figure 4A) and DD patients (Figure 4B), △ferritin levels were significantly lower in the daprodustat group than that in the placebo group (MD = −85.40, [95% CI, −126.17 to −44.64], *p* < 0.0001; MD = −141.31, [95% CI, −196.02 to −86.60], *p* < 0.00001; respectively) or in the rhEPO group (MD = −28.66, [95% CI, −33.02 to −24.30], *p* < 0.00001; MD = −82.17, [95% CI, −146.51 to −17.83], *p* = 0.01; respectively).

#### Changes in Transferrin Saturation Levels From Baseline(△Transferrin Saturation)

**FIGURE 5:**
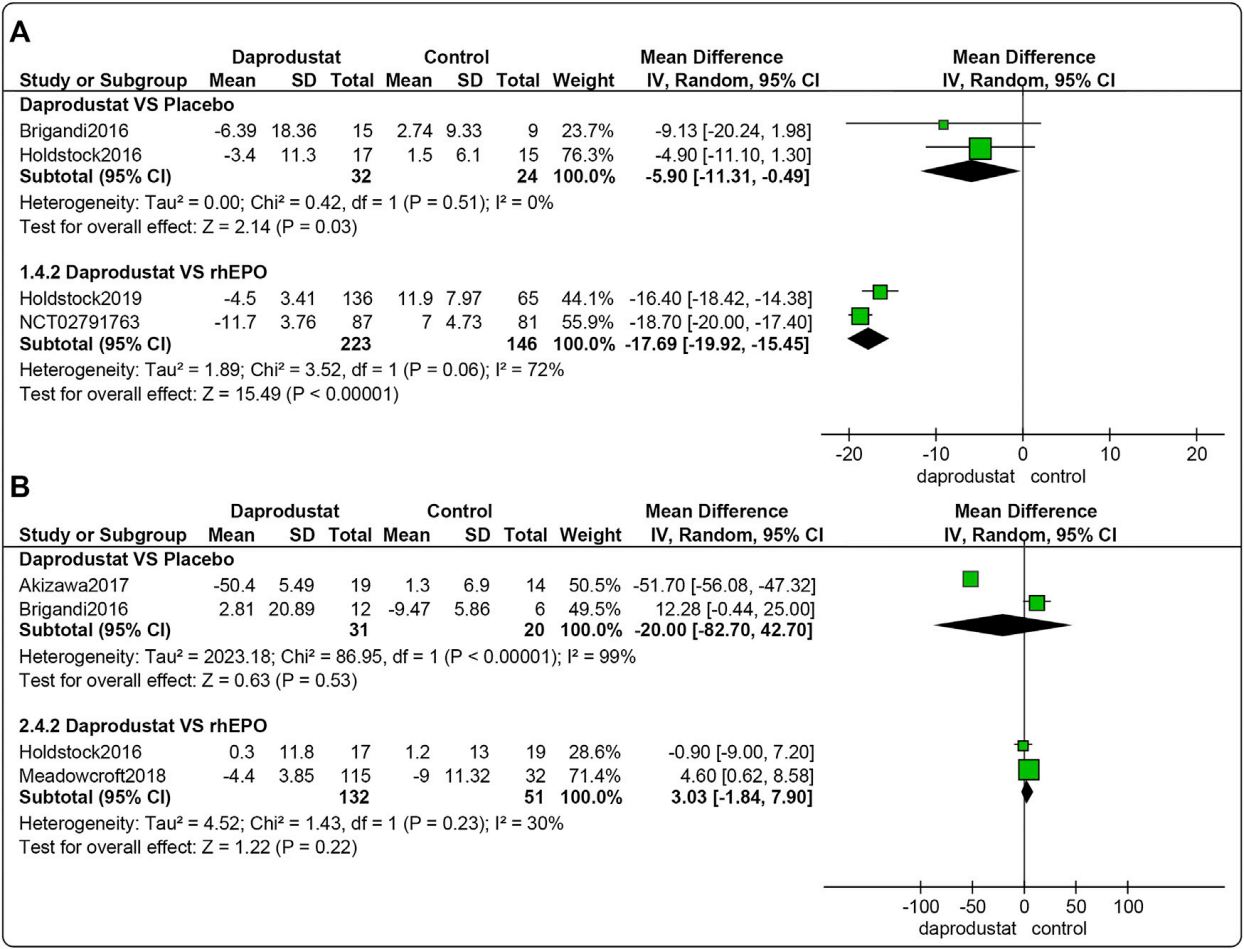
The meta-analysis results of daprodustat for △TSAT. **(A)** The pooled results for NDD patients; **(B)** The pooled results for DD patients.

Four studies (Brigandi et al., 2016; Holdstock et al., 2016; Holdstock et al., 2019; GlaxoSmithKline, 2016a) involving 425 participants reported △TSAT levels for NDD patients. Among them, two trails were comparisons between daprodustat and placebo, and two studies compared daprodustat with rhEPO. As shown in Figure 5A, △TSAT levels were significantly lower in the daprodustat cohort than in placebo or rhEPO groups for NDD patients (MD = −5.90, [95% CI, −11.31 to −0.49], *p* = 0.03; MD = −17.69, [95% CI, −19.92 to −15.45], *p* < 0.00001; respectively).

Four studies (Brigandi et al., 2016; Holdstock et al., 2016; Akizawa et al., 2017; Meadowcroft et al., 2019) involving 234 participants reported △TSAT levels for DD patients. Among these, two trials involved comparisons between daprodustat and placebo, and two studies were comparisons between daprodustat and rhEPO. As shown in Figure 5B, there was no significant difference in △TSAT levels between daprodustat and placebo or rhEPO for DD patients (MD = −20.00, [95% CI, −82.70 to 42.70], *p* = 0.53; MD = 3.03, [95% CI, −1.84 to 7.90], *p* = 0.22; respectively).

#### Changes in Total Iron-Binding Capacity Levels From Baseline (△Total Iron-Binding Capacity)

**FIGURE 6:**
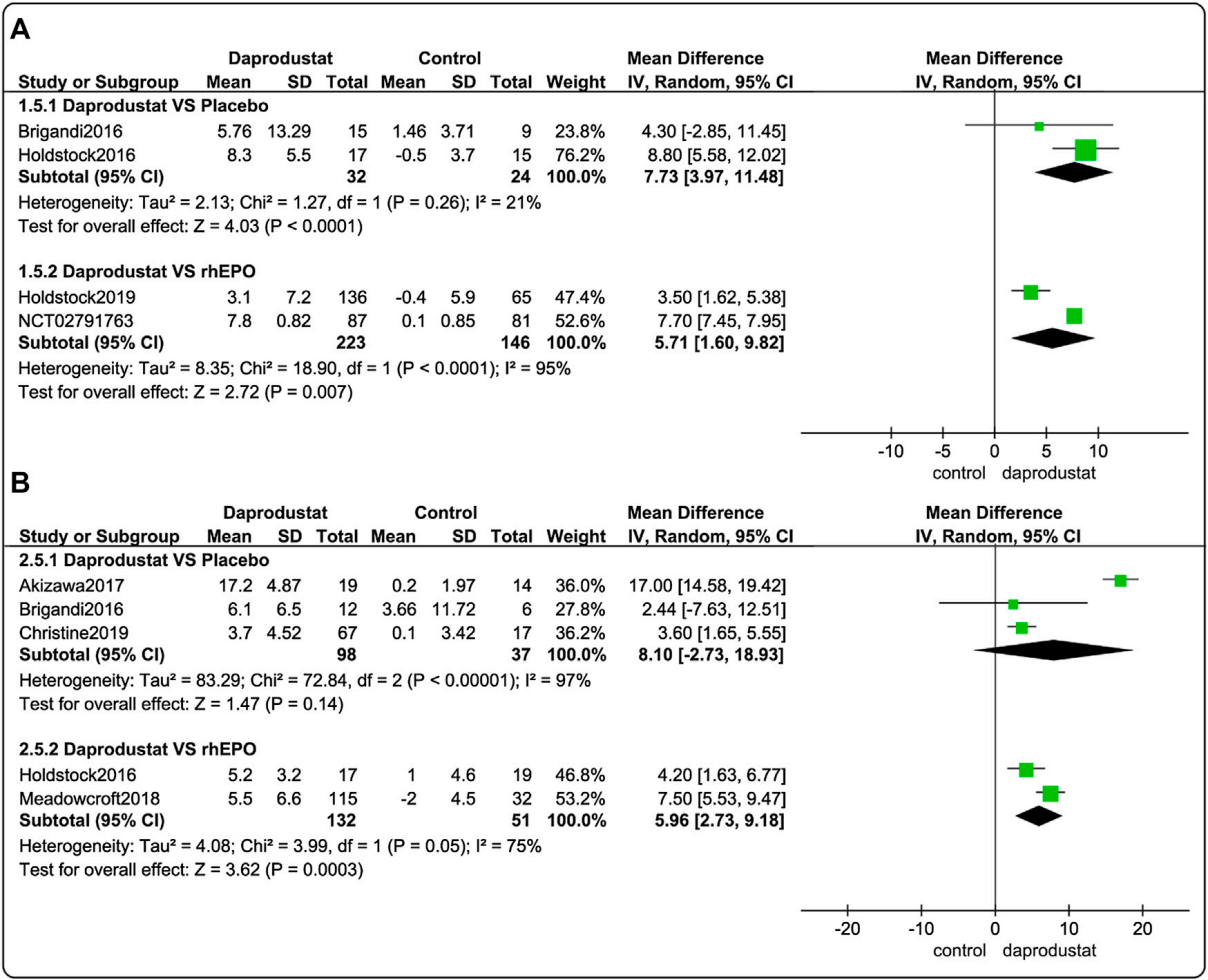
The meta-analysis results of daprodustat for △TIBC. **(A)** The pooled results for NDD patients; **(B)** The pooled results for DD patients.

Four studies (Brigandi et al., 2016; Holdstock et al., 2016; Holdstock et al., 2019; GlaxoSmithKline, 2016a) involving 425 participants reported △TIBC levels for NDD patients. Among these, two trials were comparisons between daprodustat and placebo, and two studies compared daprodustat and rhEPO. Five studies (Brigandi et al., 2016; Holdstock et al., 2016; Akizawa et al., 2017; Bailey et al., 2019; Meadowcroft et al., 2019) involving 318 participants reported△TIBC levels for DD patients. Among these, three trials were comparisons between daprodustat and placebo, and two studies compared daprodustat with rhEPO. The pooled results showed that for both NDD patients (Figure 6A) and DD patients (Figure 6B), △TIBC levels was significantly higher in the daprodustat group than that in the placebo cohort (MD = 7.73, [95% CI, 3.97 to 11.48], *p* < 0.0001) or in the rhEPO group (MD = 5.71, [95% CI, 1.60 to 9.82], *p* = 0.007; MD = 6.28, [95% CI, 4.71 to 7.84], *p* < 0.00001; respectively), while there was no significant difference in △TIBC levels between daprodustat and placebo for DD patients (MD = 8.10, [95% CI, -2.73 to 18.93], *p* = 0.14).

#### Serious Adverse Events

**FIGURE 7:**
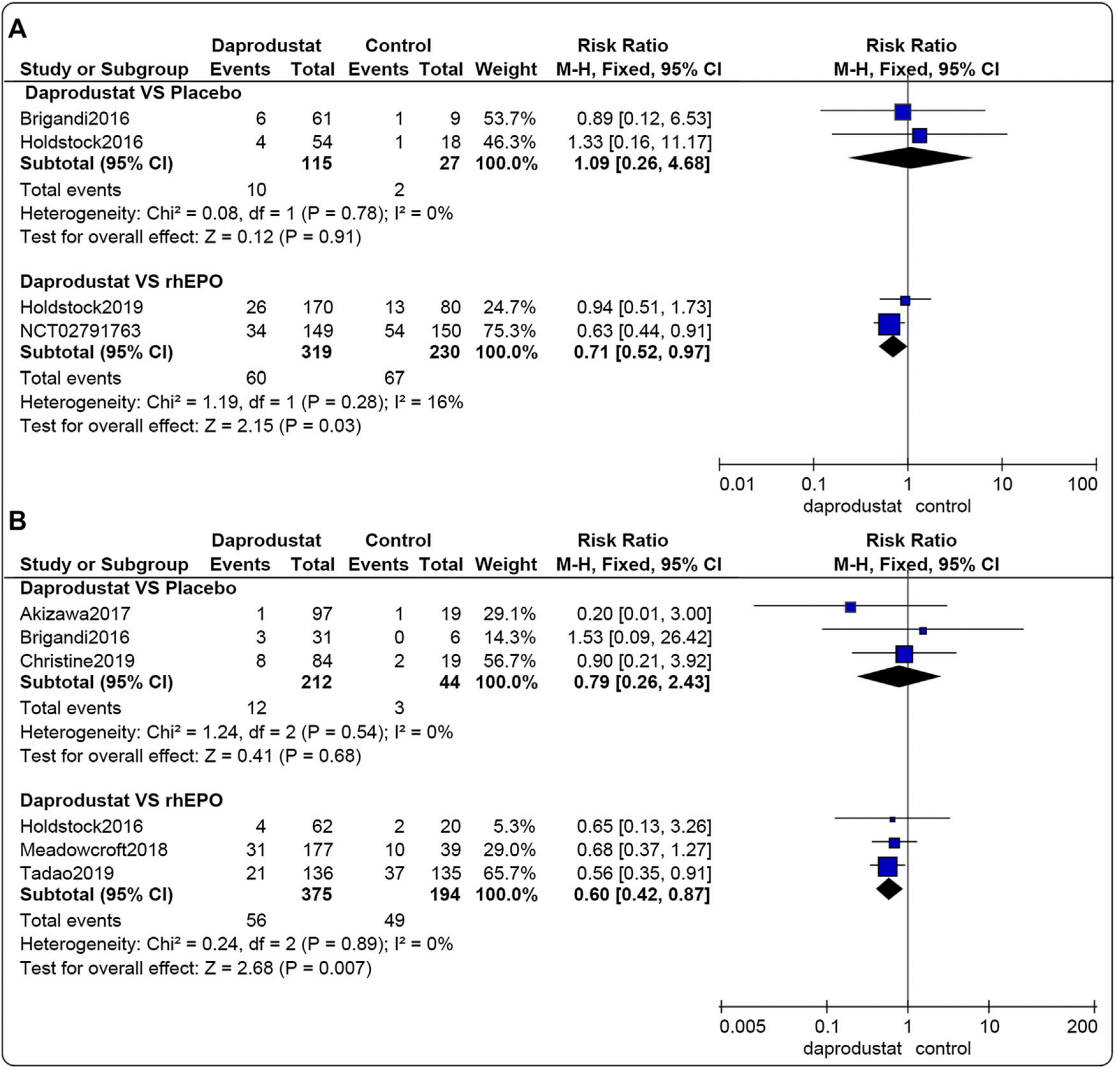
The meta-analysis results of daprodustat for SAEs. **(A)** The pooled results for NDD patients; **(B)** The pooled results for DD patients.

Four studies (Brigandi et al., 2016; Holdstock et al., 2016; Holdstock et al., 2019; GlaxoSmithKline, 2016a) involving 691 participants reported SAEs associated with NDD patients. Among these, two trials were comparisons between daprodustat and placebo, and two studies compared daprodustat with rhEPO. Six studies (Brigandi et al., 2016; Holdstock et al., 2016; Akizawa et al., 2017; Bailey et al., 2019; Meadowcroft et al., 2019; GlaxoSmithKline, 2016b) involving 825 participants reported SAEs associated with DD patients. Among these, three trials compared daprodustat and placebo, and three studies involved comparisons of daprodustat and rhEPO. The pooled results showed that for both NDD patients (Figure 7A) and DD patients (Figure 7B), there was no significant difference in the incidence of SAEs between daprodustat and placebo (RR = 1.09, [95% CI, 0.26 to 4.68], *p* = 0.91; RR = 0.79, [95% CI, 0.26 to 2.43], *p* = 0.68; respectively). Additionally, the incidence of SAEs in the daprodustat group was significantly lower than that in the rhEPO group (RR = 0.71, [95% CI, 0.52 to 0.97], *p* = 0.03; RR = 0.60, [95% CI, 0.42 to 0.87], *p* = 0.007; respectively).

#### Changes Involving of Vascular Endothelial Growth Factor

Four studies (Brigandi et al., 2016; Holdstock et al., 2016; Akizawa et al., 2017; Meadowcroft et al., 2019) reported changes in VEGF levels for NDD patients, and three studies (Brigandi et al., 2016; Holdstock et al., 2016; Holdstock et al., 2019) similarly reported for DD patients. These studies showed that for both NDD and DD patients there were considerable inter-subject variability in terms of VEGF levels; there was no trend observed in increasing plasma VEGF abundance in daprodustat-treated subjects when compared to the placebo or rhEPO subjects.

#### Publication Bias

All outcome indicators were observed for less than ten studies, so publication bias was not examined.

## Discussion

By compiling the available RCTs, our meta-analysis evaluated the current totality of evidence investigating the efficacy and safety of the novel oral HIF inhibitor, daprodustat. Overall, for both NDD and DD patients, daprodustat was associated with a superior and significant increase in Hb levels compared to placebo, and was non-inferior to parenteral rhEPO with regard to increased levels of Hb. In terms of iron metabolism, ferritin levels were significantly reduced in the daprodustat group than in the placebo or rhEPO groups for NDD and DD patients. TIBC levels were significantly increased in the daprodustat group than in the placebo or rhEPO groups for NDD patients, and it was also significantly increased in daprodustat groups when compared with rhEPO groups for DD patients. TSAT levels were significantly reduced in the daprodustat group compared to the placebo or rhEPO groups for NDD patients. However, for DD patients, notable differences in changes in TSAT levels were not detected between the daprodustat group and the placebo or rhEPO groups. Furthermore, current RCTs involving daprodustat have shown that for both NDD and DD patients, no trend of increasing plasma VEGF is observed in daprodustat-treated subjects when compared to placebo or rhEPO subjects (Brigandi et al., 2016; Holdstock et al., 2016; Akizawa et al., 2017; Holdstock et al., 2019; Meadowcroft et al., 2019). In regard to safety, the incidence of SAEs in the daprodustat group was comparable to that of the placebo group and significantly superior to that of the rhEPO group for the treatment of anemia for both NDD and DD patients. In these cases, the results were rated as providing low-quality or very low-quality evidence.

Daprodustat is a once-daily oral prolyl hydroxylase inhibitor that stimulates erythropoiesis in a manner similar to the natural response to hypoxia, whereby inhibition of HIF prolyl-4-hydroxylases (HIF-PH, PHD1, PHD2, PHD3) by daprodustat ultimately results in increased transcription of HIF-responsive genes, including erythropoietin (Johnson et al., 2014; Ariazi et al., 2017). The use of inhibitors of HIF-PH to stimulate erythropoiesis in both animal and human clinical studies has been previously described (Rosen et al., 2010; Barrett et al., 2011; Hong et al., 2013; Hong et al., 2014) and reviewed (Muchnik and Kaplan, 2011; Rabinowitz, 2013; Zhao and Wu, 2013; Kim and Yang, 2015). A missense mutation in the *HIF-α* gene was discovered in patients with familial erythrocytosis and has been found to lead to stabilization of the HIF-α protein (Percy et al., 2008). Moreover, a study showed that Hb levels increased after treatment with an inhibitor of HIF-PH, while endogenous erythropoietin remained within the normal physiological range (Flamme et al., 2014), which suggests that HIF-PHIs may use a mechanism different from ESAs to improve anemia. All of the above findings support the effectiveness of daprodustat for treatment of anemia. Our study showed that daprodustat can increase Hb levels more effectively than placebo in both NDD and DD patients, and the effect of increasing or maintaining Hb levels is not inferior to that of rhEPO.

Furthermore, HIF also regulates iron metabolism and handling (Yoon et al., 2006). HIF-2α appears to be an isoform primarily responsible for regulating iron metabolism genes in liver, with HIF-1α playing a lesser role (Kapitsinou et al., 2010). HIF-1 upregulates transferrin, ceruloplasmin, and transferrin receptor 1, the latter facilitating increased plasma transport of iron to tissues (Rolfs et al., 1997; Bianchi et al., 1999; Tacchini et al., 1999). HIF-2α boosts intestinal absorption of iron by upregulating crucial genes, including duodenal cytochrome b and divalent metal transporters 1 and 2 involved in iron uptake and export (Mastrogiannaki et al., 2009; Kapitsinou et al., 2010). According to evidence from this meta-analysis, in the NDD population, ferritin and TSAT levels were significantly decreased in the daprodustat arms, whereas TIBC increased more notably than in the placebo or rhEPO groups, suggesting that daprodustat increased iron utilization due to increased erythropoiesis in the NDD population. These results are consistent with experimental data from animal studies (Anderson et al., 2013; Wilkinson and Pantopoulos, 2013), which demonstrated that the PHD/HIF axis coordinates iron metabolism with erythropoiesis via transcriptional regulation of genes involved in iron uptake, release, and transport (Haase, 2013b). On the other hand, in studies involving DD patients, changes in ferritin levels in daprodustat arms were similar to those in NDD patients compared with control groups (both placebo and rhEPO), while there were no similar changes in terms of TSAT and TIBC. Further investigations concerning the effects of daprodustat on iron mobilization in this patient population will require larger-sample sizes and longer-term studies. The above findings will be tested in current large-sample phase three trials (ClinicalTrials.gov numbers, NCT02876835 and NCT02879305.)

In addition to modulation of EPO expression, HIF-1 also upregulates the expression of VEGF. This protein is known to stimulate angiogenesis and has been implicated in the support of tumor growth, as well as macular edema and proliferative retinopathy through its angiogenic activity (Goel and Mercurio, 2013; Fogli et al., 2016). In the present study, for both NDD and DD patients, the results demonstrated no trend of increasing plasma VEGF concentrations in the daprodustat-treated patients when compared to the placebo or rhEPO subjects. Another study involving diabetic retinopathy patients also indicated that no clinically-significant changes were observed during ophthalmologic exams, nor were any ocular events suggestive of enhanced angiogenesis identified during 24 weeks of daprodustat treatment (Tsubakihara et al., 2020). But these studies all lack long term follow-up and those with diabetic eye changes are the best to follow over several years of therapy. So, the concern that some still have about VEGF still need large, long-term follow-up clinical studies to ease.

This study analyzed the safety of daprodustat in the treatment of anemia in CKD patients. The results showed that for both NDD and DD patients, the proportion of patients with one or more SAEs was comparable to that of the placebo group, and the incidence of SAEs in the daprodustat group was significantly lower than that in the rhEPO group. Similarly, a previous meta-analysis (Zhong et al., 2018) also found no difference in adverse reactions when HIF-PH inhibitors were compared with placebo. The advantage of lower SAE incidence with daprodustat compared to rhEPO may be associated with the mechanisms involved in HIF-PH inhibitor activity in the treatment of renal anemia, which could mitigate adverse outcomes caused by exogenous high-dose EPO treatment due to the fact that they allow for physiological levels of EPO to stimulate red blood cell (RBC) production rather than high intermittent blood levels caused by the pharmacological administration of exogenous ESA. Increased cardiovascular morbidity and mortality in ESA-treated patients is associated with supraphysiologic EPO dosing and plasma EPO levels (Vaziri and Zhou, 2009; McCullough et al., 2013), HIF-PH inhibitor therapy therefore has the potential to improve cardiovascular outcomes in CKD patients (Sanghani and Haase, 2019). These findings suggest that daprodustat was well tolerated in anemic populations with CKD. Considering that HIF-PH inhibitors are involved in different pathways, publication of some recently completed or ongoing trials (ClinicalTrials.gov numbers, NCT03029208 and NCT02879305) and emergence of real-world data will further aid in determining the safety profile of daprodustat.

Potential advantages of daprodustat compared to rhEPO and its analogs in the treatment of CKD anemia include that daprodustat raises Hb levels without exposing patients to supraphysiologic EPO levels, and that daprodustat can improve iron availability for erythropoiesis, which may reduce the need for iron supplementation and avoid the need for parenteral injection (Lenihan and Winkelmayer, 2016). Current studies show that approximately 5- to 17-fold lower plasma EPO levels were measured in CKD patients who were successfully treated with HIF-PH inhibitors compared to those receiving epoetin alfa (Holdstock et al., 2016; Provenzano et al., 2016). Furthermore, preclinical studies have consistently shown that pre-ischemic HIF activation protects multiple organs from acute ischemia-reperfusion injury, including the kidneys, heart, brain, and liver (Kapitsinou and Haase, 2015). Therefore, HIF-PH inhibitors such as daprodustat are likely to become a crucial alternative tool for management of anemia in CKD patients.

However, there remain many problems in the application of daprodustat in clinical settings and in future research. Given the biology of the HIF pathway, systemic PHD inhibition has the potential to produce undesirable on-target effects ranging from changes in glucose, fat, and mitochondrial metabolism, to alterations to cellular differentiation, inflammation, vascular tone, and cell growth (Chavez et al., 2008; Lee et al., 2014). Furthermore, it is unclear whether HIF-PH inhibitor therapy may be harmful in CKD patients with certain comorbidities, such as a history of previous stroke or autoimmune disease, for example, systemic lupus erythematosus. In addition, patients may experience higher risk of SAEs when administered ESAs to achieve a higher Hb target level (Drüeke et al., 2006; Pfeffer et al., 2009), and it is still unknown whether these statements also apply to daprodustat. Future long-term trials should proceed with caution, and studies assessing daprodustat targeting Hb levels are required.

This systematic review and meta-analysis investigated the body of evidence available regarding the use of daprodustat in the treatment of anemia in CKD patients, but there were certain limitations. First, all included RCTs had only short-term results, thus we were not able to determine the long-term efficacy and possible adverse reactions of daprodustat. Second, the dosage of daprodustat in our study varied, and several phase-II studies have demonstrated that daprodustat exerts a dose-response effect on Hb levels, and a number of iron metabolism indices in both NDD and DD patients (Brigandi et al., 2016; Holdstock et al., 2016; Akizawa et al., 2017; Bailey et al., 2019). Thus, the results of this meta-analysis may be more relevant for higher daprodustat doses. Third, it should be noted that our findings are based on phase-II and phase-III studies, and the number of studies included in this study was small. Hence, the results of our study still need to be verified in several ongoing large-scale, international, phase-III studies (ClinicalTrials.gov numbers, NCT02876835, NCT03409107, and NCT02879305.)

## Conclusion

According to the current evidence, daprodustat was able to effectively increase or maintain Hb levels in both NDD and DD patients, and was non-inferior to parenteral rhEPO. The risk of SAEs with daprodustat administration was not observed when compared with placebos and was significantly lower than those in the rhEPO group. These findings support the short-term use of daprodustat for the treatment of anemia in CKD patients.

## Data Availability Statement

The original contributions presented in the study are included in the article/Supplementary Material, further inquiries can be directed to the corresponding authors.

## Author Contributions

All authors contributed to the conception, search terms and methodology of the review. QZ, YL, and WL came up with the study idea. QZ, YW, YL, and WL designed the study. YW and XF completed the database searches and study selection. YW and HY completed the assessment of bias of the included studies. XF and RW extracted data from the included studies. QZ, LS and HY completed the meta analyses. QZ and YW wrote the first draft of the manuscript. YL and WL completed the critical revision of the manuscript. All authors contributed to the writing or revision of the final manuscript. And all authors have read and approve the final version submitted to this journal.

## Funding

This research was funded by the National Natural Science Foundation of China (grant numbers 81774278 and 81373829).

## Conflict of Interest

The authors declare that the research was conducted in the absence of any commercial or financial relationships that could be construed as a potential conflict of interest.
